# SeqFusionNet: A hybrid model for sequence-aware and globally integrated acoustic representation

**DOI:** 10.1371/journal.pone.0330691

**Published:** 2025-09-08

**Authors:** Tianhao Wu, Wei Ma, Ouyuping Gu, Bing Yang, Yuheng Zhou, Jun Li

**Affiliations:** 1 College of Mechanical and Electrical Engineering, Sichuan Agricultural University, Ya’an, Sichuan Province, China; 2 College of Information Engineering, Sichuan Agricultural University, Ya’an, Sichuan Province, China; 3 Agricultural Information Engineering Higher Institution Key Laboratory of Sichuan Province, Ya’an, Sichuan Province, China; 4 Ya’an Digital Agricultural Engineering Technology Research Center, Ya’an, Sichuan Province, China; MARE – Marine and Environmental Sciences Centre, PORTUGAL

## Abstract

Animals communicate information primarily via their calls, and directly using their vocalizations proves essential for executing species conservation and tracking biodiversity. Conventional visual approaches are frequently limited by distance and surroundings, while call-based monitoring concentrates solely on the animals themselves, proving more effective and straightforward than visual techniques. This paper introduces an animal sound classification model named SeqFusionNet, integrating the sequential encoding of Transformer with the global perception of MLP to achieve robust global feature extraction. Research involved compiling and organizing four common acoustic datasets (pig, bird, urbansound, and marine mammal), with extensive experiments exploring the applicability of vocal features across species and the model’s recognition capabilities. Experimental results validate SeqFusionNet’s efficacy in classifying animal calls: it identifies four pig call types at 95.00% accuracy, nine and six bird categories at 94.52% and 95.24% respectively, fifteen and eleven marine mammal types reaching 96.43% and 97.50% accuracy, while attaining 94.39% accuracy on ten urban sound categories. Comparative analysis shows our method surpasses existing approaches. Beyond matching reference models on UrbanSound8K, SeqFusionNet demonstrates strong robustness and generalization across species. This research offers an expandable, efficient framework for automated bioacoustic monitoring, supporting wildlife preservation, ecological studies, and environmental sound analysis applications.

## Introduction

Animal sound recognition has become increasingly prominent in recent years owing to its critical importance in species conservation, biodiversity monitoring, and behavioral analysis. Compared to conventional visual observation, which is often influenced by environmental factors like distance, lighting, and obstruction, acoustic monitoring offers a more direct and efficient approach to detecting and identifying species through their vocalizations [1]. However, despite progress in artificial intelligence and signal processing, current animal sound classification models still face significant challenges, including limited generalization across species, vulnerability to background noise, and inadequate feature extraction techniques.

Traditional machine learning approaches, including Hidden Markov Models (HMM) and Gaussian Mixture Models (GMM), have been extensively employed in animal vocalization classification. Rabiner et al. [2,3] pioneered HMM for speech recognition with notable success, yet its performance diminishes under noisy conditions. Reynolds et al. [4] investigated GMM for speech emotion recognition, highlighting its capacity to represent feature distributions, though it faces difficulties with intricate and overlapping auditory sources.

Yao et al. [5] utilized GMM integrated with wavelet spectrum analysis for avian song classification, achieving 72.4% accuracy, though robustness remained constrained when addressing varied bird species.

Deep learning techniques have greatly enhanced classification performance over conventional methods. Convolutional neural networks (CNNs), recognized for their robust feature extraction capabilities, are extensively applied to audio classification tasks. Ruff et al. [6] employed a CNN to categorize 14 bird and mammal vocalizations, reaching 90% accuracy. Yin et al. [7] developed an AlexNet-inspired model for pig cough detection, achieving 96.8% accuracy. While these models deliver high precision, CNNs predominantly extract spatial features from time-frequency representations and fail to model extended temporal relationships in sequential audio signals. This shortcoming reduces their effectiveness in differentiating animal vocalizations characterized by long-term vocal patterns or sequential variations.

Recurrent neural networks (RNNs), especially gated recurrent units (GRUs) and long short-term memory (LSTM) networks, have been developed to handle the sequential characteristics of animal vocalizations. Zhang et al. [8] implemented a GRU-based classifier for four bird call types, achieving 97% accuracy. Compared to CNNs, GRUs can model temporal relationships by retaining contextual information across time, which enhances their suitability for sequential audio analysis. Nevertheless, GRUs require greater computational resources and extended training durations, especially when applied to large datasets. Furthermore, RNN-based architectures are vulnerable to gradient instability (vanishing or exploding gradients), limiting their effectiveness in analyzing lengthy audio sequences.

Transformers have recently been applied to audio classification owing to their capacity to model long-term relationships. Zhang et al. [9] adapted the Transformer encoder to extract speech and textual features, attaining 75.6% accuracy on the IEMOCAP dataset. Gong et al. [10] designed an Audio Spectrogram Transformer, achieving 98.1% accuracy on Speech Commands V2 and 95.6% accuracy on ESC-50, highlighting its dominance in general audio classification. Despite their powerful feature extraction, Transformers divide audio data into segments that risk fragmenting critical temporal structures, resulting in diminished accuracy for bioacoustic tasks.

Recent models such as AST [11] and PaSST [12] have shown promise in general audio classification; however, their effectiveness in domain-specific tasks like animal sound recognition remains less explored.

To overcome these challenges, we introduce SeqFusionNet, a new animal vocalization classification framework that merges Transformer-driven sequence encoding with MLP-guided global feature learning. This combined strategy strengthens the model’s capacity to identify both localized and overarching patterns in animal calls, enhancing generalization across species and noise robustness. In contrast to earlier methods dependent on CNNs or recurrent structures alone, our design mitigates Transformer’s segmentation limitations by integrating MLP-Mixer, enabling richer feature representations.

The key contributions of this work are outlined below:

1. We revealed the expressive potential of animal call features through varied processing and extraction approaches, and established the essential role of traditional call signal processing techniques in animal call experimentation.
2. Our work explores the best features for different species and analyzes the most effective methods to extract features across animal types, establishing a crucial foundation for subsequent animal studies.
3. We introduce SeqFusionNet, a novel network structure with robust global perception for animal vocalizations. This design strengthens feature representation, mitigates Transformer’s segmentation shortcomings, and prioritizes simplicity and efficiency, allowing deployment in more intricate scenarios.
4. We conducted extensive experiments using a variety of datasets, including pig, bird, marine mammal, and urban sound datasets, to evaluate the performance of SeqFusionNet. The results demonstrate the superiority of our method in terms of accuracy and generalization.
5. Comparisons with current methods spanning multiple species (birds, pigs, marine mammals) validate SeqFusionNet’s superior performance across datasets, where interspecies differences exert minimal influence, positioning it as a robust and scalable solution for bioacoustic classification.

## Related work

Hidden Markov Model (HMM) is a model for solving sequence annotation problems with powerful time series modeling capabilities and easy portability of recognition results [2]. In the 1980s, Rabiner L R et al. first applied HMM to the field of speech recognition and achieved good results, since then HMM technology has been widely used in the field of speech recognition [3]. The speech recognition system designed by Maseri M et al. uses MFCC feature extraction algorithm in the front end and defines HMM recognition in the back end, the overall system performance accuracy is high [13].

Gaussian Mixture Model (GMM) is a speech emotion recognition model that describes the statistical distribution of speech emotion feature parameters through a linearly weighted superposition of Gaussian probability density functions [4]. GMM is widely used in the fields of image recognition and speech recognition, etc. Yao W et al. used GMM to fit spectral images and extract model parameters expressing acoustic features, and proposed a feature fusion method based on wavelet spectra of GMM and MFCC to realize the recognition based on birdsong [5]. These methods typically depend on handcrafted features and may struggle to scale across diverse and noisy real-world datasets.

However, these traditional approaches heavily rely on simplified statistical assumptions. Their generalization capacity is limited when faced with complex, noisy, or large-scale acoustic environments, which motivates the use of more powerful deep models in our study.

Convolutional Neural Network (CNN) is one of the representative algorithms of deep learning - it is a feed-forward neural network with special structure [14]. With the continuous improvement and deepening of deep learning theory, Convolutional Neural Networks have been widely used in the fields of natural language, image, [15] speech, video, etc. Eledath D et al. proposed a multi-temporal-frequency (t-f) resolution CNN architecture for end-to-end speech recognition from raw speech waveforms, and the performance of the proposed multi-t-f architecture gained in comparison with the multiscale feature-based system and SincNet. -f architecture performance is gained [16]. Ganapathy S et al. processed the time, frequency, and channel dimensions of the input spectrum and utilized a convolutional layer to learn the representations and proposed a three-dimensional (3-D) convolutional neural network (CNN) architecture for multichannel far-field ASR, showing improvements over the baseline system [17]. Passricha V et al. proposed a hybrid CNN-BLSTM architecture that facilitates a continuous speech recognition task [18]. However, CNNs are inherently limited in modeling long-range dependencies in temporal sequences.

In recent years, deep learning has rapidly advanced and found widespread application across diverse fields, including license plate and face recognition, speech processing, recommendation systems, and autonomous driving. Notably, it has also led to significant progress in animal vocalization classification. Zhao J. proposed a DNN-HMM-based acoustic model for continuous pig cough recognition and demonstrated that its word error rate (WER) was consistently lower than that of the traditional GMM-HMM system [19]. While DNNs have shown improved performance over GMM-based acoustic models in hybrid architectures, GMM-HMM systems still dominate many speech recognition applications. Meanwhile, CNNs and RNNs have demonstrated promising results and are increasingly adopted for acoustic modeling tasks [20].

Yin Y proposed an algorithm for classifying pig coughs based on a fine-tuned AlexNet model and spectrogram features, and the proposed algorithm is significantly better compared to the performance of probabilistic neural network classifiers and some existing algorithms, with cough and overall recognition accuracies of 96.8% and 95.4%, respectively [7]. Madhusudhana S et al. proposed a combined CNN and LSTM model for the recognition of fin whale (Balaenoptera physalus) sounds, which reduced the performance variance with respect to the baseline CNN model, resulting in an increase in the area under the exact-memory curve by 9-17%, and an increase in the peak F1 score by 9-18% [21]. However, these models are often tailored to specific species, with limited testing on multi-species or multi-environmental datasets.

In addition, since being proposed in the field of natural language processing, Transformer has also shown superior performance in the field of speech recognition [22]. Dong L proposed the Speech-Transforme model and a two-dimensional attention mechanism, and the best model achieved a word error rate (WER) of 10.9% and the whole training process was significantly faster than the cyclic sequence-to-sequence model [23]. Nevertheless, Transformers may lack strong inductive biases for capturing local time-frequency patterns, which can affect their sensitivity to subtle acoustic variations.

The key issue in speech emotion recognition is the extraction of speech emotion features, and the extraction of speech emotion features determines the results of speech emotion recognition [24]. T Ilion et al. extracted 35-dimensional rhyming features from EMO-DB corpus with 51% recognition accuracy [25]. Y H Kao et al. extracted rhyming features from frame, syllable, and word level respectively to classify four emotions with 90% recognition accuracy [25]. Upadhya S S et al. proposed an improved Mel-scale filter bank based speech emotion recognition method with 6.3% accuracy improvement over the traditional MFCC filter bank [26]. Chowdhury A et al. combined MFCC and LPC features to solve the problem of recognition from heavily degraded audio data, thus improving the recognition performance in challenging situations [27]. Chauhan N et al. combined LPC, MFCC, and ZCR features with FFANN and SVM classifiers to conduct a comparative study of various feature combinations for speech recognition systems and found that the efficiency of the system did not degrade due to the increase in the number of samples when using a combination of LPC, MFCC, and ZCR features with ANN classifiers [28]. Shen W proposed a new fusion feature MFCC-CNN and analyzed the effect of different number of fused frames on the classification performance and found that softmax and SVM classifiers have the best performance by fusing 55 and 45 adjacent frames respectively. It can be concluded that the speech recognition task is heavily dependent on the effectiveness of speech feature extraction [29].

In summary, while existing studies have made substantial progress in leveraging various neural architectures for bioacoustic and environmental sound classification, there remains a lack of integrated models that can simultaneously capture local spectral patterns and long-range temporal dependencies. In this study, we first compare several commonly used acoustic feature representations and select the most effective one for our downstream task. To address the remaining limitations, our proposed SeqFusionNet introduces a dual-branch design that effectively combines Transformer and Mixer modules, aiming to unify the strengths of prior methods within a single framework.

## Materials

### Pre-processing of vocalisation signals

In this study, we collected sound data from a variety of animals (pigs, birds, marine mammals). The duration of one of the sound samples from the pig was canonical and we did not need to consider processing his sound duration. But for birds as well as marine mammals, the data samples are not canonical. This is due to the fact that for the recognition model, the input features need to maintain the same dimensional size, and need to retain the complete information in the input features, and too large a length of data can easily lead to too much arithmetic power in the training process of the model. Similarly, too small a data sample will result in less information being retained in the features, which will directly lead to poorer model recognition ability. Therefore, we constrain the length to to standardize it in terms of retaining complete feature information as well as feature dimensions. The bird call data contains a total of about 820 bird calls with a duration of about 2s or 3 minutes. Considering the impact of data irregularities, we decided to constrain the call lengths, and we processed each category individually and divided each call data into normalized data with a duration of 2s. In subsequent processing, the divided data were filtered and voiceless samples were excluded to ensure that the data samples contained bird call information, resulting in a total of 11,670 bird song calls in the dataset. In the treatment of marine mammals, we used the same method for processing, and obtained a total of 1,648 data samples, and the above data set is the supporting data set of this paper. Considering that the animal call is a non-smooth time-varying signal and contains low-frequency background noise in the sound samples, in order to eliminate this effect, we pre-processed the obtained call samples with pre-emphasis, frame-splitting and windowing [30].

As the sound emitted by an animal is influenced by factors such as glottal excitation and lip-oral radiation, the power of the signal significantly attenuates as frequency increases. This attenuation causes the vocalisation signal to exhibit a high signal-to-noise ratio (SNR) at low frequencies and a low SNR at high frequencies. To compensate for this, pre-emphasis is applied to enhance the high-frequency components of the vocalisation signal, thereby flattening its spectrum. This adjustment ensures a more uniform SNR across the frequency band, which facilitates spectral analysis. T We feed the vocalisation signal into a first-order high-pass filter for processing, where the high-pass filter is calculated as shown in Equation 1 below.

$$H(z)=1-\alpha z^{-1}$$ (1)

Here, *H*(*z*) is the transfer function of the filter in the z-domain, ${\text{z}}^{-1}$ is the unit delay operator, and *α* is the pre-emphasis coefficient (typically set to 0.95).

Subsequent pre-emphasis on the sound normalized with respect to the normalized sound yields, calculated as shown in Equation 2 below.

$$x_{p}(n)=x_{n}(n)-\alpha x_{n}(n-1)$$ (2)

In this equation, *x*_*n*_(*n*) and $x_{n}(n\;-\;1)$ are the current and previous samples of the normalized signal, respectively, and *x*_*p*_(*n*) is the pre-emphasized output.

Generally speaking, vocalisation signals exhibit non-smooth temporal patterns due to rapid changes in frequency and amplitude. To reduce temporal variability and ensure local stationarity within a frame for subsequent processing, we compress the duration of such calls into a relatively short window (e.g., 40–80 ms), so that the signal within each frame can be approximated as quasi-stationary. To preserve continuity between adjacent frames, overlapping is applied during framing. The overlap is determined by the difference between the frame length and the frame shift. The calculation of the sub-frame is described in Equation 3 below.

$$f_{n}=\frac{N-\text{overlap}}{\text{inc}}=\frac{N-\text{wlen}}{\text{inc}}+1$$ (3)

N denotes the signal length, inc is the data frame shift length, wlen is the short-time frame length, and overlap represents the overlap between adjacent frames. In the subsequent processing, each processed frame signal is multiplied by a window function to smooth signal transitions and reduce spectral leakage. We specifically choose the Hamming window for this purpose. The Hamming window attenuates the edges of each frame to reduce side lobe leakage, thereby preserving spectral integrity and improving the accuracy of frequency domain analysis. By applying this windowing process, we mitigate artificial distortions introduced by abrupt frame boundaries, ensuring a well-defined spectral representation across the frequency range. The calculation of the window function is shown in Equation 4 below

$$f(x)=\left\{ \begin{array}{cc} 0.54-0.46\cos\left(\frac{2\pi n}{L-1}\right), & 0\leq n<L-1\\ 0, & \text{else} \end{array}\right.$$ (4)

Preprocessing the animal calls helps standardize the data and reduces the computational complexity, enabling more efficient model computation

### Feature extraction

The selection of features is of great significance to the computation of the model, in this paper, in order to fully demonstrate the characteristics of animal call data, we use eight speech feature extraction methods, which contain chroma_cens (computing chroma energy normalization), chroma_stft (computing chromaticity map), melspectrogram (computing spectrogram in Mel scale), mfcc (computes the Mel frequency), Spectral_contrast (computes the spectral contrast used for vocalisation signals), Spectral_cqt (computes the constant Q chromaticity diagram), Tonnetz (computes the tonal center-of-mass feature), and Poly (obtains the coefficients of the nth-order polynomial fit to the columns of the spectrogram), which are eight features that are used by the Librosa library [31]. The extraction is performed and the features extracted by this feature extraction method will be the main features used in this paper.

### Dataset

In this paper, four datasets are used: the pig sound dataset, bird sound dataset, marine mammal sound dataset, and UrbanSound8K [32]. Among them, the pig sound dataset was collected from a farm, where sound data were recorded from five feeder pigs under four different states: calm, feeding, frightened, and anxious. The dataset contains a total of 1,746 recordings, which were gathered firsthand from the feedlot environment. The bird data was downloaded from the open-source xeno-canto website, and a total of 9 different species were downloaded. Due to the differences in the duration of each audio file, we cropped the data obtained from, and a total of 11,670 call samples were compiled, which were stratified and divided into training, validation, and testing sets using a ratio of 7:2:1. To avoid data leakage, we ensured that recordings from the same individual or session did not appear across multiple subsets. Due to the large amount of categories of data and the number of samples, the data could not be processed by any other means. The marine mammal call data contains a total of 15 different marine species such as whales, seals, etc., and was obtained from the Watkins Marine Mammal Sound Database. Our normalization of the data yielded a total of 3,296 data samples, which were stratified and divided into training, validation, and test sets in the same manner. Again, individual and session-level separation was maintained. These data were obtained from the Watkins Marine Mammal Sound Database. The UrbanSound dataset was obtained from the URBAN-SED Dataset, which contains 8,732 annotated sound clips, each recording is about 4s in length and contains 10 different urban environment sounds such as air conditioning sound, car honking, etc. After normalizing the data, we applied the same stratified splitting and ensured no recording-level overlap between subsets. In this work, we use these four different datasets for classifier testing, and the specific division of each type of dataset is shown in Table 1 below:

**Table 1:** Distribution of different species and segmentation of the dataset.

Class	Train	Val	Test	Total
Bird (9)	8169	2334	1167	11670
Marine Mammals (15)	2306	660	330	3296
Pig (4)	2800	800	400	4000
Ubsound (10)	6113	1747	873	8733
Total	19388	5541	2770	27699

## Methods

### SeqFusionNet model structure

**Fig 1:**
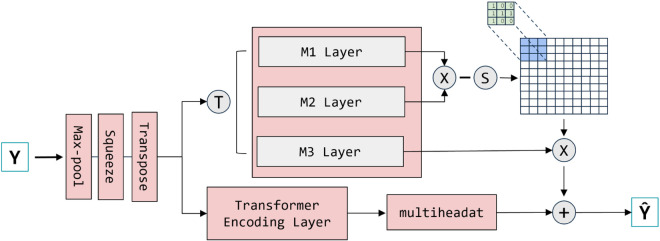
Schematic diagram of SeqFusionNet structure.

Guo et al. proposed a self-attentive module called TA for capturing temporal information in 2021, while in the same year, Xia et al. introduced the use of MLP for modeling contextual dependencies. Inspired by these approaches, we address the information loss problem in Transformer-based animal call segmentation by integrating both Transformer and MLP-Mixer mechanisms [33,34]. In this paper, we propose SeqFusionNet, a hybrid model that combines MLP-Mixer and Transformer for feature extraction and classification, using MFCC features as input, as illustrated in Fig 1. The model consists of two key components: an MLP-Mixer module for global feature extraction and a Transformer-based encoder for contextual feature modeling. The MLP-Mixer consists of three layers (M1, M2, and M3), where M1 and M2 compute feature weight interactions, and their outputs are further processed by M3 to capture comprehensive global information. The other module is built on a Transformer architecture, retaining a Transformer encoder to effectively capture sequential dependencies However, instead of using a complete Transformer decoder, it incorporates a local multi-head attention mechanism after the encoder to further refine the learned representations. The final output embeddings from both the MLP-Mixer and Transformer components are fused for classification. This hybrid structure effectively compensates for the segmentation-based information loss in Transformer models, ensuring both global feature perception and local contextual awareness in animal call classification.

As shown in Fig 1, we compute the weights W1 computed by M1 and M2 with M3 to get a new global perceptual information, and on the other side, we obtain the global information of the features through the Encoding layer of Transformer, which is then fed into the Multihead Attention Mechanism to focus on the more detailed feature importance of the features, and finally merge the two to get the final output. It is worth noting that SeqFusionNet computes two different global features at the same time and constrains the feature information of the MLP by weight computation, while preserving the computed features of the Transformer, which we hope to use to enhance the expressiveness of the features in this way. We have simplified the specific implementation structure in this model, which will be explained in detail during the experiments.

### Detailed design description of SeqFusionNet

**Fig 2:**
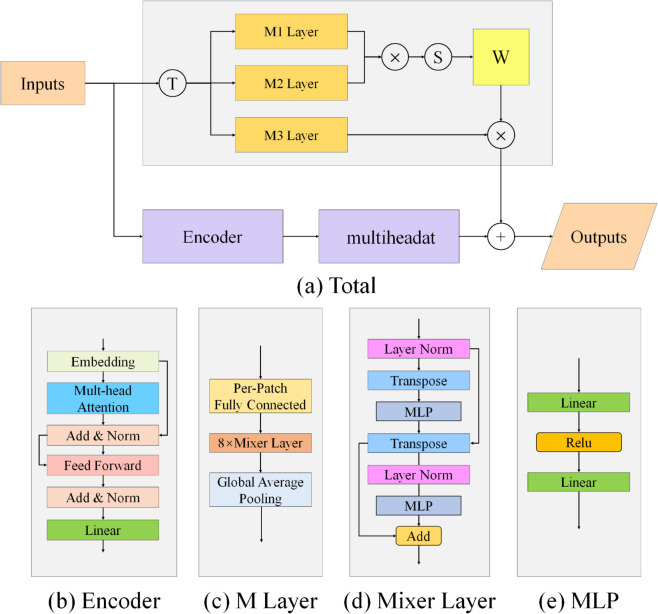
Detailed design diagram of SeqFusionNet.

In this paper, we propose an animal call classification model named SeqFusionNet, whose network structure is shown in Fig 2, which contains two different modules in total, of which Transformer is the main structure, in which we consider the specificity of animal calls, i.e., the model doesn’t have to consider the ability of linguistic expression, so we only use the Encoding structure for global feature extraction, in Encoding’s multi-head attention structure, the sound data is equally distributed to multiple heads by segmentation, which may lose the capture of information at the sound segmentation. This is due to the fact that the vovalisation signal is a kind of temporal information, which is characterized by its temporal sequence, and the information may be lost by the direct segmentation. Therefore, we use the MLP in another module for global temporal information extraction, which strengthens the global extraction capability of the model and does not lose the information due to the segmentation. The specific model structure and the structure of each module are shown in Fig 2.

As shown in Fig 2 above, a is the complete SeqFusionNet structure, b is the Encoder structure of the Transformer, c is the structure of the M layer, d is the structure of the Mixer layer, e is the structure of the MLP. We designed an animal call classification model called SeqFusionNet, which takes 60x60 F1 features (MFCC) as input, and firstly, the input MFCC features are passed through a layer of Maxpooling to make the feature points in the neighborhood take the maximum value, which is used to reduce the error caused by the shift of the estimated mean value due to the parameter error in the subsequent layer, and thus retain more feature information. The processed features are then fed into the MLP layer, which contains three perceptual models, M1, M2, and M3, the input features of these three models are the same, but the difference is that the output features of M1 are matrix multiplied by the outputs of M2, and the output probability matrix W is outputted through a softmax function, which is then multiplied by the probability matrix W and M3.

In the subsequent calculation, the result will be used as the global feature gain and merged with the output features of the Transformer to obtain the feature F1 with a stronger global signal. By this way of design, the global sensing ability of the model can be effectively enhanced on the basis of retaining the advantages of the model, and in addition, it also compensates for the loss of neighboring timing signals brought about by the Transformer segmentation. The model is designed in such a way that it can effectively enhance the global perception capability of the model while retaining the advantages of the model.

### MLPmixer model structure and presentation

Multi-layer perceptrons (MLPs) are powerful models for approximating nonlinear functions, as they efficiently aggregate information from previous layers. The strength of MLPs comes from their ability to fuse information through linear layers, allowing each subsequent layer to process features that integrate information from earlier steps. In this process, each dimension of the feature in the next step encompasses the information from the previous step. This approach eliminates the need for spatial or temporal location awareness, which reduces the model’s sensitivity to the specific arrangement of the input features. However, since MLPs require a fixed input structure, the MFCC features must be transformed or reshaped to fit this input format.

The module contains three hidden layers, each of which is a linear layer, and a RELU is added after each layer as an excitation function for monitoring useful features. At the same time, we reduce the data jitter during training by normalizing the data using LayerNorm to ensure the training speed of the model. By adding the MLPmixer module, we can obtain richer feature information while ensuring the training speed of the model.

**Fig 3:**
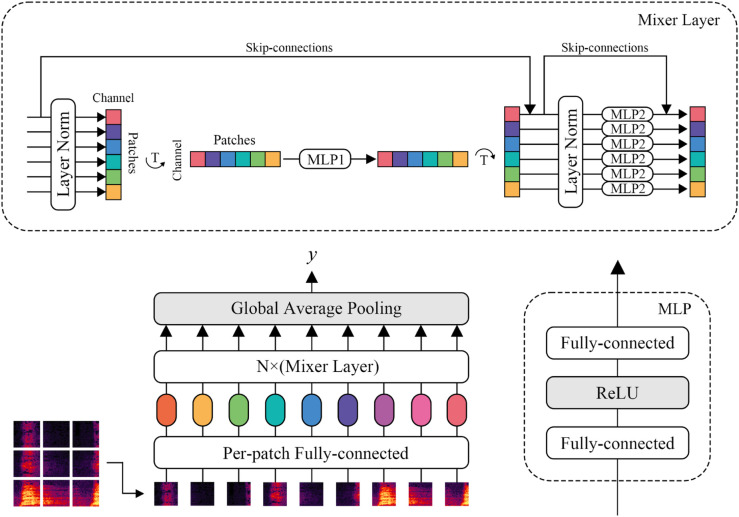
Schematic diagram of the MLPmixer module.

The Mixer architecture allows the MLP to also show excellent local feature learning capability, as shown in Fig 3, we first split the input audio features into multiple patches and feed them into the Mixer Layer, and the token mixing MLP in the Mixer architecture allows the model to mix features on different patches, and since the input features have only one channel, the role of channel mixing MLP in Mixer becomes to enhance the perception of features on a single patch. These two types of layers are executed alternatively to facilitate the interaction of information between dimensions. In addition, we use skip-connections similar to the ResNet structure to prevent gradient vanishing during training and for feature enhancement.

### Transformer model structure and introduction

The Transformer model was originally introduced for natural language processing tasks, particularly machine translation, using an encoder-decoder architecture [22]. While earlier time-series models like RNNs and LSTMs demonstrated strong performance, they are inherently limited by their fixed time-step processing, making it difficult to capture long-range dependencies effectively. Considering these limitations, we opted to use the Transformer model for this task.Unlike RNN-based models, which process sequences sequentially, the Transformer leverages self-attention mechanisms to capture global dependencies across the entire sequence simultaneously. This capability is particularly beneficial for animal sound classification, where relevant acoustic patterns may span long time intervals. Since our task does not require sequence generation, we employ only the Transformer’s encoder structure, omitting the decoder to focus purely on feature extraction and classification.

**Fig 4:**
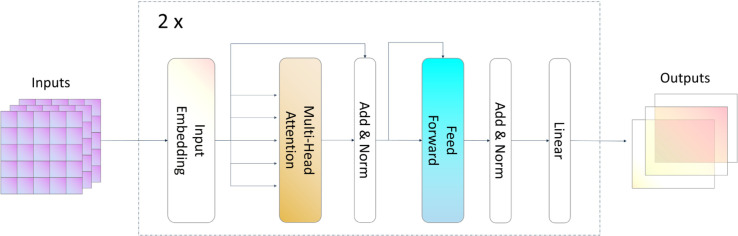
Schematic diagram of the transformer module.

As shown in Fig 4 above, we use a two-layer encoder layer. This module takes MFCC as input features and first encodes the input features to feed into the attention layer, we use a five-headed attention machine mechanism for computing global features and at the end use a linear layer to impose constraints on the features so that the output dimensions of the model are consistent with the inputs and easy to operate with the MLP structure. By using a multi-head attention mechanism for detaching the drawbacks of the traditional sequence model, the model’s ability to extract global information is enhanced, and it is not easy to receive the influence of the time step to get richer holographic information.

### Multi-attention mechanisms

Multi-head attention mechanisms play a key role in the model to learn different behaviors based on the corresponding attention mechanisms, each head’s attention mechanism focuses on the local importance of the feature or global information, and then combines the different behaviors as knowledge to capture the various ranges of dependencies within the sequences, and when dealing with the features, the multi-head attention mechanisms are able to focus on them in a fine-grained way by calculating the attention weights for the importance of different locations.

**Fig 5:**
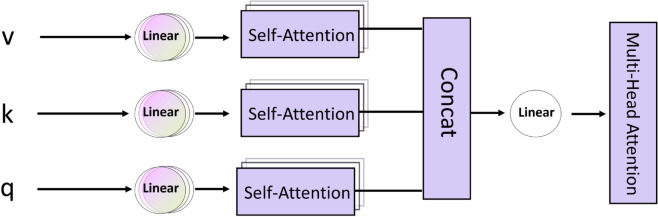
Schematic diagram of the multiple attention mechanism.

As shown in Fig 5, we use the output via the transformer-encoder as input to the multi-head attention mechanism, which linearly transforms the features through a self-attention mechanism, where each attention head can focus on different feature subspaces, and then integrate the information from these subspaces. This feature fusion helps to extract richer and more useful representations, which improves the performance of the model, and then the outputs of the different heads are aggregated for the purpose of balancing the attention to global information and local importance, and finally we perform the transformation by another linear projection that can be learned to produce the final output.

Since the transformer-encoder is fed into the feed-forward neural network for nonlinear transformation and mapping from the shallow features through a layer of multi-head attention mechanism, we believe that there is a certain lack of attentional attention of the model to the key features, which can lead to the loss or redundancy of the feature information, so we retain a layer of multi-head attention mechanism after the structure to pay more attention to the deeper features of the global importance, avoiding the model to deeply learn global information about irrelevant features.

### Training equipment and parameter setting

During the hyperparameter configuration process, we considered common practices from deep learning models in acoustic classification tasks and adjusted them based on the specific characteristics of the datasets used in this study. An initial set of parameters was selected through grid search on a validation set comprising 10% of the data. Key parameters including learning rate, batch size, and number of epochs were tuned to achieve an optimal trade-off between classification accuracy and training stability.

To ensure a fair comparison, all models including SeqFusionNet and the baseline architectures were trained using the same configuration. We employed the Adam optimizer, which is well-suited for non-stationary objectives and sparse gradients. The initial learning rate was set to $1\times 10^{-3}$, and L2 weight decay was applied at a value of $5\times 10^{-4}$ to prevent overfitting. A total of 100 training epochs were performed, with early stopping based on the validation loss and a patience of 20 epochs to avoid unnecessary training. The batch size was set to 32 to balance computational efficiency with generalization performance.

To further stabilize training and enhance convergence, we adopted a cosine annealing learning rate schedule, which gradually reduces the learning rate to fine-tune the model parameters during later epochs. A dropout rate of 0.2 was applied throughout the network to mitigate overfitting. The complete set of training hyperparameters is summarized in Table 2.

**Table 2:** Unified training parameters used across all models.

Parameter	Value
Optimizer	Adam
Initial Learning Rate	$1\times 10^{-3}$
Weight Decay	$5\times 10^{-4}$
Batch Size	32
Number of Epochs	100
Early Stopping Patience	20
Learning Rate Scheduler	Cosine Annealing
Dropout Rate	0.2

## Results

### Experiments

In this study, we initially utilized Mel-frequency cepstral coefficients (MFCCs) to perform a preliminary evaluation of the model’s performance and characteristics. MFCCs are widely used features that exhibit strong general applicability and have demonstrated effectiveness across various audio-related tasks.

We first extracted basic features from the dataset, including pre-emphasis and short-time Fourier transforms (STFT). The STFT decomposes the audio signal into a series of frequency components, some of which contain meaningful species information, while others may represent noise. The frequency axis is then mapped to the Mel scale, followed by the application of a discrete cosine transform (DCT) to compute the MFCCs, providing a compact representation of the audio signal.

These MFCCs are subsequently fed into a neural network, which learns to identify which patterns are relevant to the species and which ones correspond to noise, effectively extracting meaningful information from the superimposed waveforms.

By using MFCCs, we conducted a preliminary assessment of the performance of different models. Experimental results show that the Transformer model outperforms others, followed by the MLP, with the specific results presented in Table 3. To ensure the reliability of the evaluation, all experiments were conducted over five independent runs with different random seeds, and the results are reported as the average values along with standard deviations.

**Table 3:** Comparison of ACC of different models on each species.

Model	Pig (4)	Bird (9)	Marine Mammals (15)	Ubsound (10)
CNN	66.12%	54.17%	62.94%	60.76%
RNN	59.86%	52.96%	49.39%	58.35%
LSTM	68.49%	58.79%	46.06%	60.06%
GRU	72.62%	53.61%	54.36%	59.04%
MLP	67.74%	62.59%	64.28%	64.87%
Transformer	**78.12%**	**75.10%**	**89.15%**	**83.18%**

To ensure the reliability of the evaluation, all experiments were conducted over five independent runs with different random seeds. The results shown in Table 3 represent the average values across these runs. We observed that the performance fluctuations were minimal, with standard deviations less than 0.5% for all models.

As shown in Table 3, a single Transformer model achieves excellent performance, demonstrating its strong adaptability to the animal call classification task and its generalization across different datasets. Therefore, we selected Transformer as the backbone of our model. Notably, the MLP structure also performs well in this task. This is because, in the barking dataset, the sounds exhibit strong temporal continuity, which is reflected in the consistency of barking signals. The MLP network, with its powerful ability to learn feature correlations, can effectively capture these patterns without being significantly affected by external factors such as model structure or the physical properties of signals. To validate the effectiveness of our method, we employed eight different feature extraction techniques to analyze three types of animal calls and urban environmental sounds. This feature set captures diverse representations of audio information. By using this approach, we aimed to minimize the impact of individual features on the model’s performance. The extracted features include chroma_cens, chroma_stft, melspectrogram, MFCC, Spectral_contrast, Spectral_cqt, Tonnetz, and Poly. We employed the Transformer model as the backbone network to evaluate these eight acoustic features. Additionally, we reduced the number of categories in the bird and marine mammal datasets to assess the Transformer’s sensitivity to category variations while minimizing the influence of extraneous factors. The segmented data retained its original partitioning structure to ensure consistency. Experimental results revealed that the Transformer achieved superior performance, particularly with the melspectrogram and MFCC features. Moreover, its performance improved further when the number of categories was reduced. These findings highlight the effectiveness of the Transformer model in addressing the challenge of animal call classification.

We conducted extensive experiments on eight extracted features to identify the most suitable ones for the call classification task. Our findings revealed that melspectrogram and MFCC consistently outperformed the others, proving their effectiveness for this application. As a result, we retained these two features—melspectrogram and MFCC—for subsequent experiments. As shown in Table 4, the Transformer model delivers impressive performance with these features, achieving high recognition accuracy. However, its performance varies significantly with other features, primarily due to their smaller dimensions. Given the large number of animal categories in our dataset, these features yield sparse valid information, placing substantial demands on Transformer’s ability to capture essential details. This variability arises from Transformer’s dependence on segmented data, which can fragment the temporal continuity critical to animal calls. By contrast, multilayer perceptrons (MLPs)cite48 excel in global perception, efficiently aggregating information across all dimensions without segmentation-related losses, thus enhancing feature expressiveness. To capitalize on this strength, we propose SeqFusionNet, a model that merges Transformer’s sequence coding with MLP’s powerful global feature extraction to address these limitations effectively.

**Table 4:** Transformer ACC on different datasets are compared.

Hallmark	Pig (4)	Bird (9)	Marine Mammals (15)	Bird (6)	Marine Mammals (11)	Ubsound (10)
chroma_cens (12x60)	72.78%	52.45%	47.14%	56.83%	61.67%	39.24%
chroma_stft (12x60)	70.11%	33.99%	58.64%	49.15%	60.25%	41.30%
melspectrogram (50x60)	**87.79%**	71.15%	78.64%	77.56%	87.91%	73.57%
mfcc (60x60)	85.63%	**89.98%**	**89.15%**	**91.83%**	**91.87%**	**83.18%**
Spectral_contrast (7x60)	76.72%	71.18%	69.15%	50.24%	69.50%	31.01%
Spectral_cqt (12x60)	58.12%	57.50%	58.03%	53.59%	75.18%	41.30%
Tonnetz (6x60)	60.63%	54.23%	50.45%	50.59%	50.30%	30.66%
Poly (2x60)	51.45%	46.12%	42.94%	44.05%	48.40%	-

Building on the synergy between Transformer’s sequence modeling and MLP’s global perception, we developed SeqFusionNet to optimize animal call classification using the robust melspectrogram and MFCC features. The MLP component mitigates Transformer’s segmentation-induced information loss by providing a cohesive global view, while Transformer ensures precise temporal encoding. In this paper, we conducted experiments based on these two high-performing features, opting for a single layer of SeqFusionNet. This choice reflects the moderate dimensionality of our selected features, which strikes a balance between richness and complexity, making a single-layer structure sufficient to handle the task efficiently. The experimental results, detailed in Table 5, demonstrate SeqFusionNet’s exceptional accuracy and generalization across multiple datasets, underscoring its ability to overcome Transformer’s shortcomings while leveraging the strengths of both approaches.

**Table 5:** Comparison of SeqFusionNet using two features on different datasets.

Hallmark	Pig (4)	Bird (9)	Marine Mammals (15)	Bird (6)	Marine Mammals (11)	Ubsound (10)
mfcc (60x60)	**95.00%**	**94.52%**	**96.43%**	**95.24%**	**97.50%**	**94.39%**
melspectrogram (50x60)	93.33%	79.03%	91.42%	79.39%	89.29%	84.66%

As shown in Table 6, we conducted experiments between six different datasets based on two different features using SeqFusionNet, and the experimental results show that the network structure is feasible for dealing with the task of animal call classification, and the recognition accuracy reaches more than 94% for all three animals. It is worth noting that the recognition accuracies of 97.50% and 96.43% for whales, 95.24% and 94.52% for birds, and 94.39% for the urban environment sound classification task can be achieved, which shows that the structure has a strong applicability and generalization ability, and the differences between different species can be attenuated by modeling. Furthermore, the supplementary materials include per-class precision and other related metrics, which demonstrate that the model maintains strong discriminative ability across all individual categories.

**Table 6:** Comparison between SeqFusionNet and different models.

Model	Pig (4)	Bird (9)	Marine Mammals (15)	Bird (6)	Marine Mammals (11)	Ubsound (10)
SeqFusionNet	**93.33%**	**94.34%**	**96.43%**	**96.09%**	**97.50%**	**94.39%**
ResNet18	90.39%	90.16%	87.79%	91.72%	90.16%	89.02%
RNN	78.97%	67.05%	80.45%	69.20%	83.78%	83.29%
GRU	89.80%	32.24%	64.24%	52.45%	73.78%	75.86%
LSTM	76.14%	51.24%	71.06%	63.15%	81.64%	83.41%
ANN	81.37%	80.18%	72.24%	68.23%	76.95%	83.18%
Transformer	87.79%	89.98%	89.15%	91.30%	91.87%	83.18%
PaSST	85.55%	78.94%	76.95%	91.42%	93.50%	88.11%
AST	85.87%	64.09%	92.92%	72.68%	93.29%	75.14%

In order to better demonstrate the advancement of the model, we compare our proposed model structure with numerous temporal models as well as CNN models, and in order to avoid the influence of features, we use MFCC, which has the best generalization, as the input feature of the model, as the experimental results show that our proposed model structure has optimal performances on various datasets, and the recognition accuracies are higher than those of the existing models.

In addition to conventional deep learning baselines like ResNet18, RNN, and GRU, we further benchmarked SeqFusionNet against recent state-of-the-art Transformer-based models, including PaSST and AST, which have shown strong results in general audio classification tasks. Notably, while AST and PaSST attain competitive performance on selected datasets (e.g., AST achieves 92.92% on Marine Mammals (15)), their accuracy fluctuates significantly across tasks (e.g., dropping to 64.09% on Bird (9) for AST and 76.95% on Marine Mammals (15) for PaSST). In contrast, SeqFusionNet achieves over 94% across all datasets, reflecting greater consistency and adaptability across diverse acoustic domains.

These results emphasize SeqFusionNet’s superior stability and scalability compared to both traditional models and advanced Transformer architectures. This robustness makes it well-suited for real-world bioacoustic applications where acoustic variability and species diversity pose major challenges.

As shown in Table 6 above, the proposed SeqFusionNet structure has excellent performance on a variety of datasets. SeqFusionNet has excellent generalization ability for the animal call classification task, and maintains excellent performance for different animal call data, thanks to the powerful global feature extraction ability of SeqFusionNet. This also proves that MLP can compensate for the drawbacks of Transformer’s feature segmentation by using MLP to effectively enhance the expressiveness of the features, and the model structure has little effect on the animal species. In addition, we compared SeqFusionNet with other models, and SeqFusionNet outperformed the existing models and showed strong performance.

Moreover, compared with conventional deep learning models like ResNet18, RNN, and GRU, SeqFusionNet consistently outperforms in every dataset with a notable margin. For instance, while ResNet18 achieves strong results with accuracy ranging from 87.79% to 91.72%, SeqFusionNet surpasses it with accuracy exceeding 94% across all datasets. Similarly, models such as LSTM and ANN exhibit instability, with accuracy dipping as low as 51.24% in challenging datasets like Bird(9). In contrast, SeqFusionNet demonstrates remarkable stability, which reinforces its adaptability to varying data distributions and species diversity. In order to visualize the differences between the models more, we present the results of our experiments as shown below.

**Fig 6:**
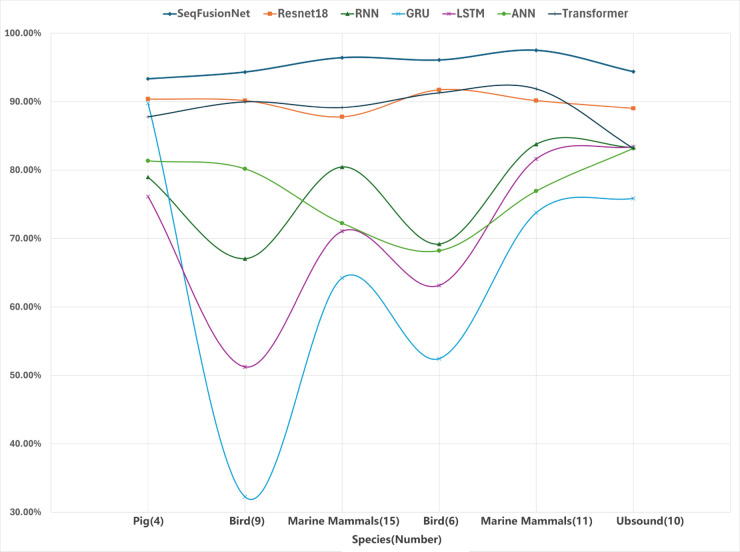
Schematic of the accuracy of SeqFusionNet on different datasets.

As shown in Fig 6 below, we compare the classification performance of multiple models, including MLPmixer-Transformer, ResNet18, RNN, GRU, LSTM, ANN, and Transformer, across different species’ datasets. The results highlight differences in model performance and generalization capabilities. The MLPmixer-Transformer achieves the most stable and superior performance across all species datasets, consistently maintaining accuracy above 90%. This validates the strong generalization capability of SeqFusionNet, as mentioned earlier, which effectively extracts global features and overcomes the segmentation issues of Transformer features.

Species datasets such as Pig(4) and Ubsound(10) are comparatively easier tasks, where all models perform relatively well. However, datasets with birds and marine mammals exhibit greater classification challenges, amplifying the differences in model robustness and generalization. ResNet18 exhibits consistently high accuracy, slightly lower than MLPmixer-Transformer. This confirms its ability to handle animal call classification tasks effectively, though it may lack some of the feature expressiveness provided by MLP-enhanced SeqFusionNet. Both RNN and GRU display moderate performance with significant variability across species. Their accuracy decreases in datasets with high intra-class variability, such as Bird(9) and Marine Mammals(15), indicating challenges in capturing complex temporal or global features.

**Fig 7:**
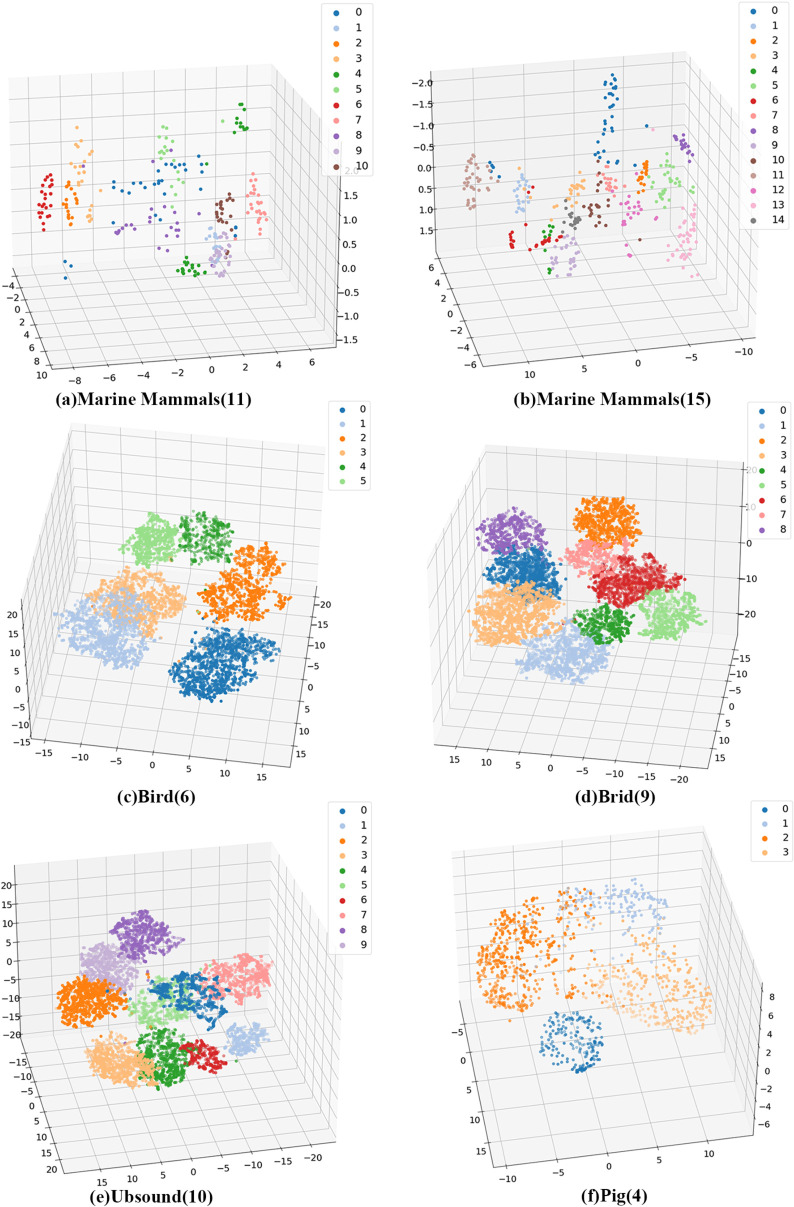
Examples of original and augmented images.

In the subsequent work, in order to visualize the recognition accuracy of the model, we used t-SNE to visualize the features of the six datasets, and we chose to use the last layer of the features as our visualization data, as shown in Fig 7 below, where we demonstrate the 3D t-SNE classification results of the model features.

As shown in Fig 7, we visualized the learned feature representations of SeqFusionNet on six datasets using the t-SNE algorithm. For this visualization, we set the perplexity to 20 and the learning rate to 200 to ensure a meaningful separation of feature clusters. The x- and y-axes represent the two-dimensional projections produced by t-SNE, which reflect the similarity structure of the original high-dimensional feature space. The resulting visual clusters indicate that SeqFusionNet is able to capture salient and discriminative patterns in the input, successfully grouping samples from the same class while maintaining clear boundaries between different classes. This provides further evidence of the effectiveness of the MLP-Mixer component in enhancing the Transformer’s global context modeling, ultimately contributing to the model’s superior performance across diverse datasets.

### Computational efficiency and model complexity

To quantitatively assess the computational efficiency and complexity of SeqFusionNet and baseline models, we measured their parameter count, FLOPs, inference latency, and memory footprint under consistent experimental settings. Specifically, all models were profiled using a single NVIDIA RTX 3090 GPU and an Intel Xeon Silver 4210 CPU. SeqFusionNet comprises approximately 7.8 million parameters and requires 1.12 GFLOPs per forward pass. The average inference time for a single audio sample (60-frame spectrogram) is 4.2 ms on GPU. These results demonstrate that SeqFusionNet provides strong performance while maintaining a reasonable computational cost.

For comparison, the Transformer-only model contains 6.4M parameters and 0.95 GFLOPs, while the Mixer-only variant (depth 4) contains 5.7M parameters and 0.89 GFLOPs. Both show slightly faster inference times but at the cost of reduced accuracy, as detailed in the ablation study.

Regarding memory usage, SeqFusionNet maintains stable GPU memory consumption of approximately 420 MB per batch of 32 inputs. Even when evaluating longer recordings using overlapping windows (e.g., 10-second segments at 50% overlap), the memory footprint increases linearly and remains manageable due to the model’s moderate size and efficient batching.

In summary, SeqFusionNet achieves a favorable trade-off between predictive accuracy and computational efficiency, making it well-suited for scalable and resource-aware acoustic analysis tasks (Table 7).

**Table 7:** Comparison of parameter count, FLOPs, inference latency, and memory footprint across models.

Model	Params (M)	FLOPs (G)	Latency GPU (ms)	Memory (MB)
SeqFusionNet	7.8	1.12	4.2	420
Transformer-only	6.4	0.95	3.8	400
Mixer-only	5.7	0.89	3.2	360
PaSST (small variant)	88.2	9.6	7.2	510
AST (base)	87.8	10.5	6.8	517
ResNet18	11.7	1.8	4.6	390
GRU	3.3	0.46	2.9	270
LSTM	3.5	0.51	3.1	290
ANN	1.5	0.19	2.1	250

### Ablation study

To validate the design of SeqFusionNet, we conducted a series of ablation experiments to evaluate the impact of individual components and hyperparameter settings, including the inclusion of Transformer and MLP-Mixer branches, the depth of the Mixer sub-network, and the temporal patch size used in the Mixer module. The results, summarized in Table 8, provide the following insights.

**Table 8:** Ablation study on model components and hyperparameters. Accuracy (%) is reported on Ubsound (10 classes) and Bird (9 classes) datasets.

Model Variant	Depth	Patch Size	Ubsound Acc (%)	Bird Acc (%)
Transformer Only	–	–	83.18	89.98
Mixer Only	4	15	83.29	91.24
SeqFusionNet	2	15	87.80	92.65
SeqFusionNet	3	15	91.98	93.26
SeqFusionNet	4	15	**94.39**	**94.34**
SeqFusionNet	8	15	92.23	92.85
SeqFusionNet	4	5	89.80	92.65
SeqFusionNet	4	10	92.23	92.66
SeqFusionNet	4	20	91.39	91.64

First, we observe that both the Transformer-only and Mixer-only variants achieve competitive performance, with the Mixer-only model slightly outperforming the Transformer-only counterpart. This indicates that the Mixer architecture is capable of capturing useful feature representations on its own. However, the full SeqFusionNet model, which integrates both structures, significantly improves accuracy on both datasets, confirming the complementary strengths of the two branches.

We further explored the effect of varying Mixer depth from 2 to 8 layers. While shallow configurations (e.g., depth = 2) exhibit limited modeling capacity, and very deep configurations (e.g., depth = 8) may suffer from overfitting and reduced generalization, the 4-layer configuration offers the best trade-off between complexity and accuracy. This suggests that a moderate depth allows the model to learn rich but generalizable representations.

In addition, we examined the impact of temporal patch size, which controls the time span aggregated into each token. A smaller patch size (e.g., 5) enables the model to focus on fine-grained temporal patterns, which is beneficial for capturing brief acoustic events. On the other hand, larger patches (e.g., 20) may blur local details, hindering accurate classification. Our results show that a patch size of 15 provides the best overall performance, striking a balance between capturing local detail and preserving global context.

These findings support the effectiveness of SeqFusionNet’s architectural choices and provide practical guidance for configuring future hybrid models for bioacoustic classification.

## Discussion

In our work, we have innovatively proposed a fusion model based on MLPmixer and Transformer for animal call classification task. In the process of this task, we have collected and organized a dataset of animal calls from a variety of animals and pre-processed the dataset to obtain a standardized data set. In addition, in order to ensure that different animals are not affected by a single feature, we also explored the adaptability of feature extraction to different animals, hoping to discover the optimal features for each animal, reduce the impact of features on the model, and highlight the recognition ability of the model. In the structure of SeqFusionNet, we used MLP for extracting global features and fused them with Transformer encoded features, MLP features strongly compensate the problems caused by Transformer cuts, and this enhancement method can be used in other similar tasks. In previous work, often only one method or one feature is used, which may not be applicable for many different categories of species. In this paper, we have demonstrated that complementary approaches can make the model more efficient, which can be used as a new idea for other scholars in the future.

We performed experimental validation using a variety of datasets containing three different species as well as UrbanSound8K. The experimental results show that the recognition accuracy of 95.00% was achieved for four states of pigs, 94.52% for nine bird species, 96.43% for fifteen marine mammals, and 94.39% for ten urban environments, which shows that the model has an excellent performance with excellent generalization for the task of animal call recognition. We compared the method proposed in this paper with the studies of other scholars. We describe the other studies in Table 9, including the team that conducted the study, the animals studied, the methodology used, and the accuracy of the model recognition.

**Table 9:** Comparison of using SeqFusionNet with other studies.

Study	Number of Classes	Methodologies	Performance
Yanling Yin et al. [7]	pigs Category 2	Spectrogram, AlexNet	ACC:96.8%
Weizheng Shen et al. [35]	pigs Category 2	SVM	ACC:97.35%
Weizheng Shen et al. [29]	pigs Category 2	MFCC-CNN	ACC:97.72%
Xie and Zhu [36]	bird species Category 14	CNN	F1-Score:95.95%
Küçüktopçu et al. [37]	bird species Category 21	minimum distance classifier	ACC:72%
Silvester Dian Handy Permana et al. [35]	bird species Category 2	CNN	ACC:96.45%
Roop Pahuja et al. [38]	bird species Class 8	MLP	P:84.5%, R:82.6%
Jiang et al. [39]	Cetaceans Category 2	CNN	ACC:95%
Marek et al. [40]	Cetaceans Category 11	HMM, GMM	ACC:84.11%
Tao Lu et al. [41]	Cetaceans Category 3	AlexNet	ACC:99.96%
Our Approach	pigs Category 4	SeqFusionNet	ACC:95.00%
Our Approach	bird species Class 9	SeqFusionNet	ACC:94.52%
Our Approach	Cetaceans Category 15	SeqFusionNet	ACC:96.43%
Our Approach	Ubsound Category 10	SeqFusionNet	ACC:94.39%

As shown in Table 9 above, we compare our proposed method with existing state-of-the-art studies, demonstrating the advantages of SeqFusionNet for different animal call classification tasks. In particular, for the study of domestic pig, our experimental results are lower than the existing studies, this is because the dataset used in this paper is more complex, other scholars only classified whether it is a coughing sound or not, in our data feeding and fearing are highly similar, this is the main reason for the low recognition results, while in other studies comparison, we used more categories of animal species, but we still maintain the excellent performance, which is an advantage due to the SeqFusionNet structure. We used two different dimensions of features to fuse them to achieve better performance, which also provides a new way of thinking about the problem of poor recognition accuracy of a single model.

It cannot be ignored that there are still limitations to this study. In this paper, we focus on the recognition task through the calls made by the animals, but for some species, there may be more significant visual differences between animals. Second, in this study, we used an ideal dataset, and in the wild, birds have a rich variety of categories and may have species in non-recognition categories, which undoubtedly for increases the difficulty of recognition. In future work, we hope to introduce visual methods into this study, and by supplementing them with vision, we can obtain richer biological information and achieve a more comprehensive and stable recognition task.

## Conclusion

In this paper, we propose a SeqFusionNet Recognition Network for the animal call classification task, which can support future animal call classification tasks. Second, we explore the applicability of different classes of features for different animals, and we show the most suitable feature extraction method for each animal. In our experiments, we compare our proposed method with currently used methods, and SeqFusionNet achieves better recognition results compared to a single model structure. In the discussion, we compare our experiments with current experiments of the same type, demonstrating the superiority and applicability of the method. This study covers the data of common animal calls, and the results of this study can provide a reference for the future classification of animal calls, as well as a new idea for other scholars’ research. In future work, this study will explore more effective methods to solve the limitations in the current work.

## Supporting information

S1 Fig Confusion matrix heatmap for the bird_6 dataset. (TIF)

S1 File Per-class precision, recall, and F1-score for the bird_6 dataset. (CSV)

S2 Fig Confusion matrix heatmap for the bird_9 dataset. (TIF)

S2 File Per-class precision, recall, and F1-score for the bird_9 dataset. (CSV)

S3 Fig Confusion matrix heatmap for the Marine Mammals_11 dataset. (TIF)

S3 File Per-class precision, recall, and F1-score for the Marine Mammals_11 dataset. (CSV)

S4 Fig Confusion matrix heatmap for the Marine Mammals_15 dataset. (TIF)

S4 File Per-class precision, recall, and F1-score for the Marine Mammals_15 dataset. (CSV)

S5 File Per-class precision, recall, and F1-score for the UrbanSound8K 10-class subset. (CSV)

S5 Fig Confusion matrix heatmap for the UrbanSound8K 10-class subset. (TIF)

S6 Fig Confusion matrix heatmap for the pig_4 dataset. (TIF)

S6 File Per-class precision, recall, and F1-score for the pig_4 dataset. (CSV)
