# Supplementary figures and images for: SeqFusionNet: A hybrid model for sequence-aware and globally integrated acoustic representation
Source: PLoS One. 2025 Sep 8;20(9):e0330691. doi: 10.1371/journal.pone.0330691 (PMC12416843; doi:10.1371/journal.pone.0330691)

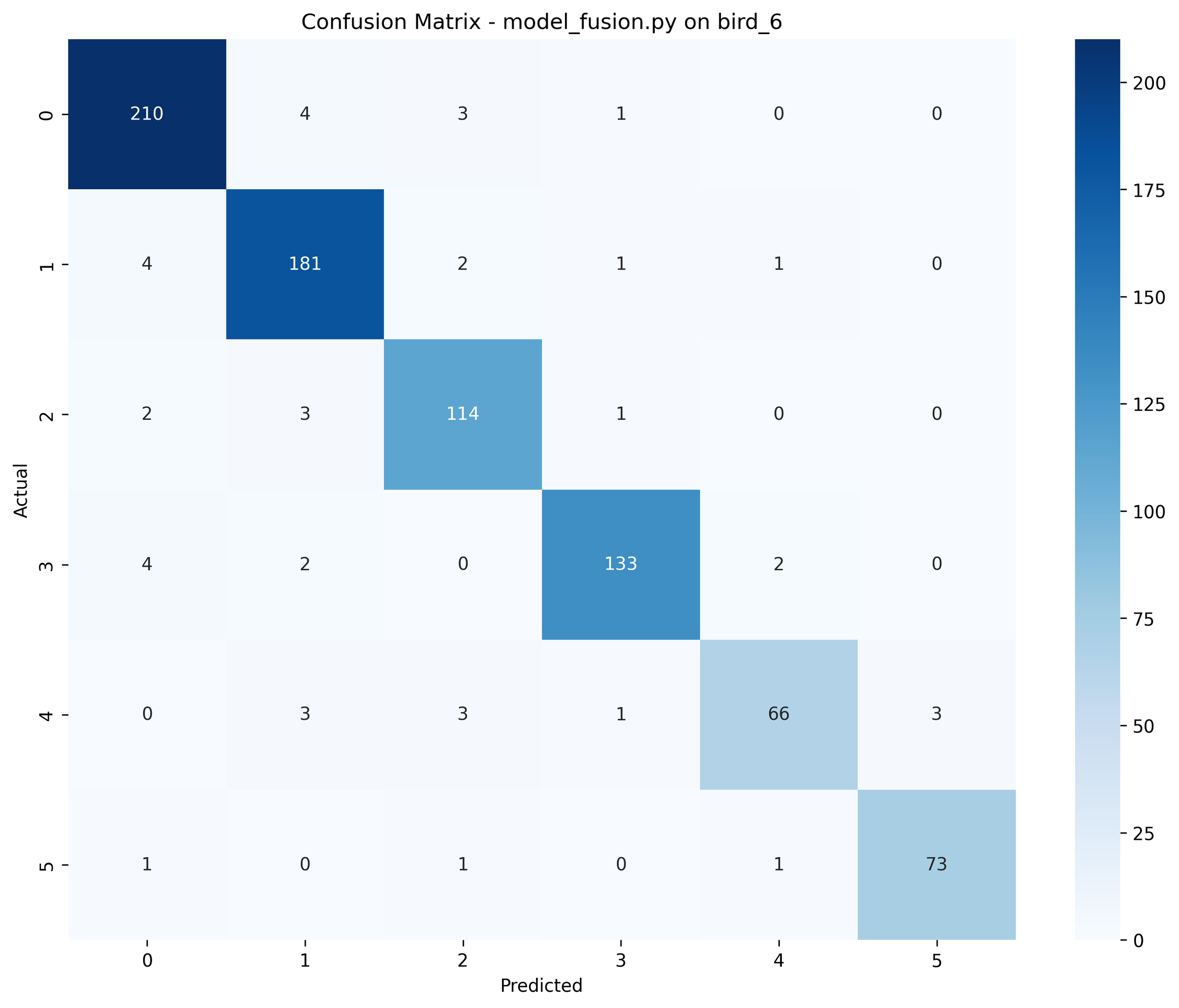

Supplement: S1 Fig — (TIF) [file pone.0330691.s001.tif]

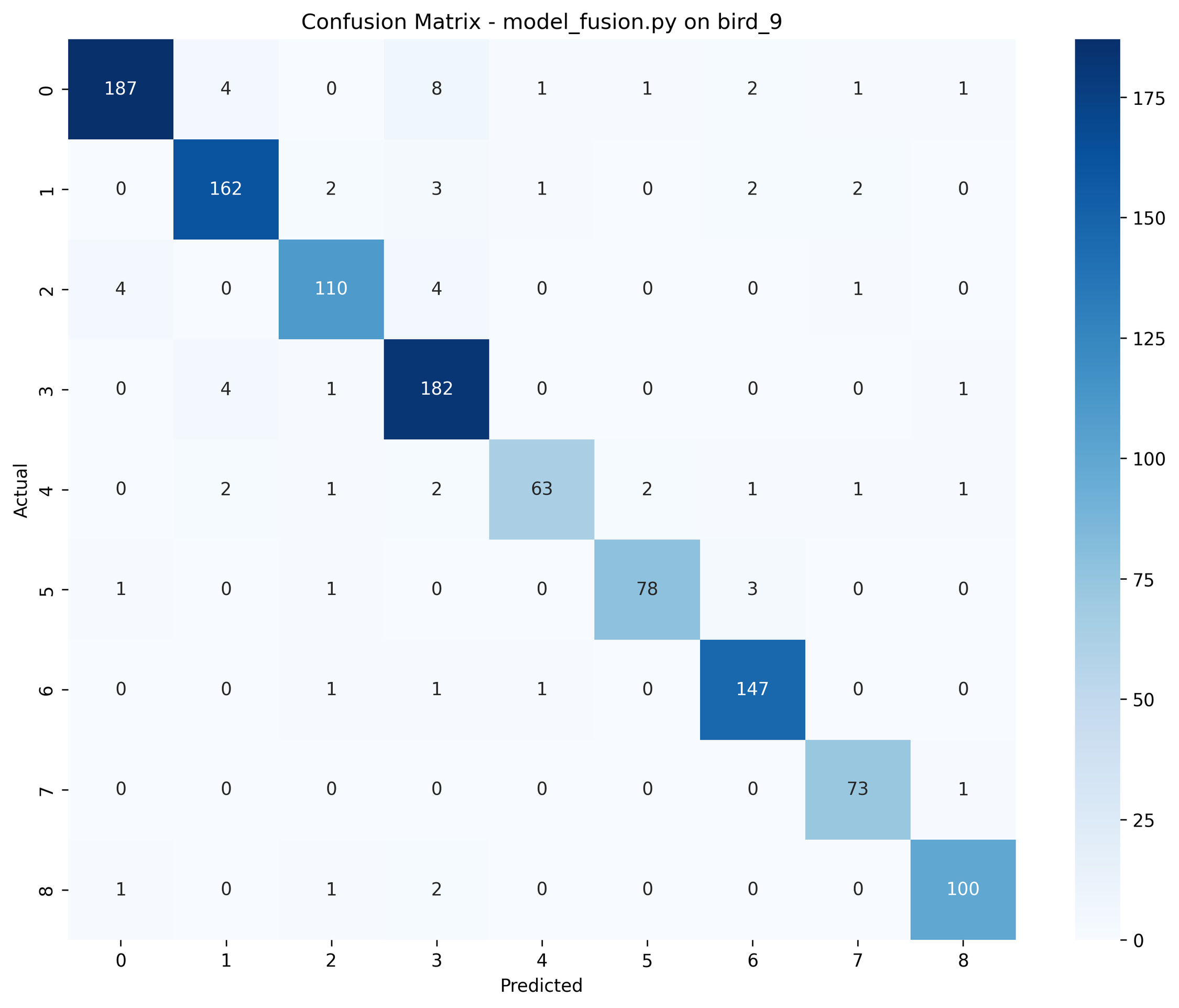

Supplement: S2 Fig — (TIF) [file pone.0330691.s003.tif]

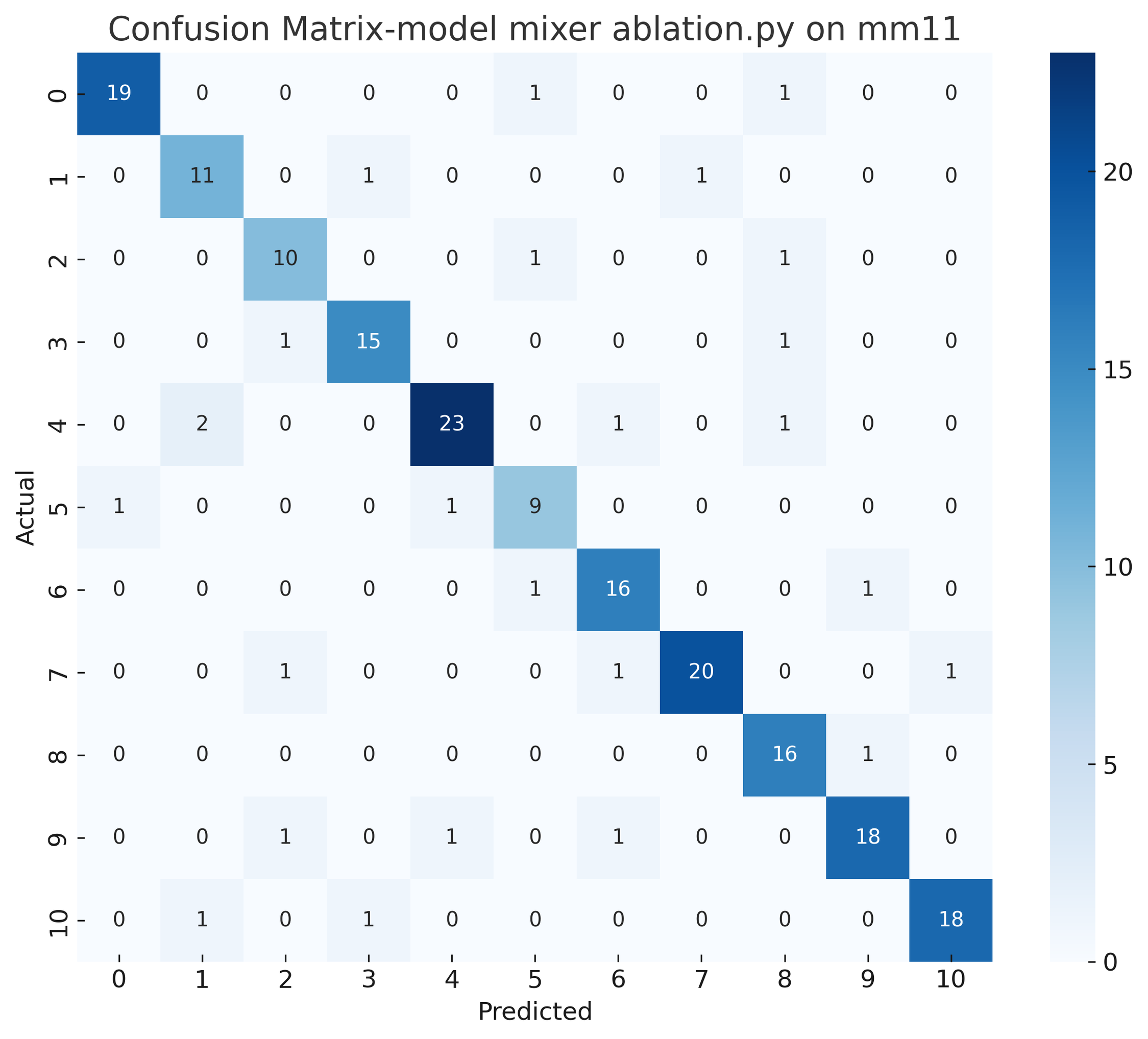

Supplement: S3 Fig — (TIF) [file pone.0330691.s005.tif]

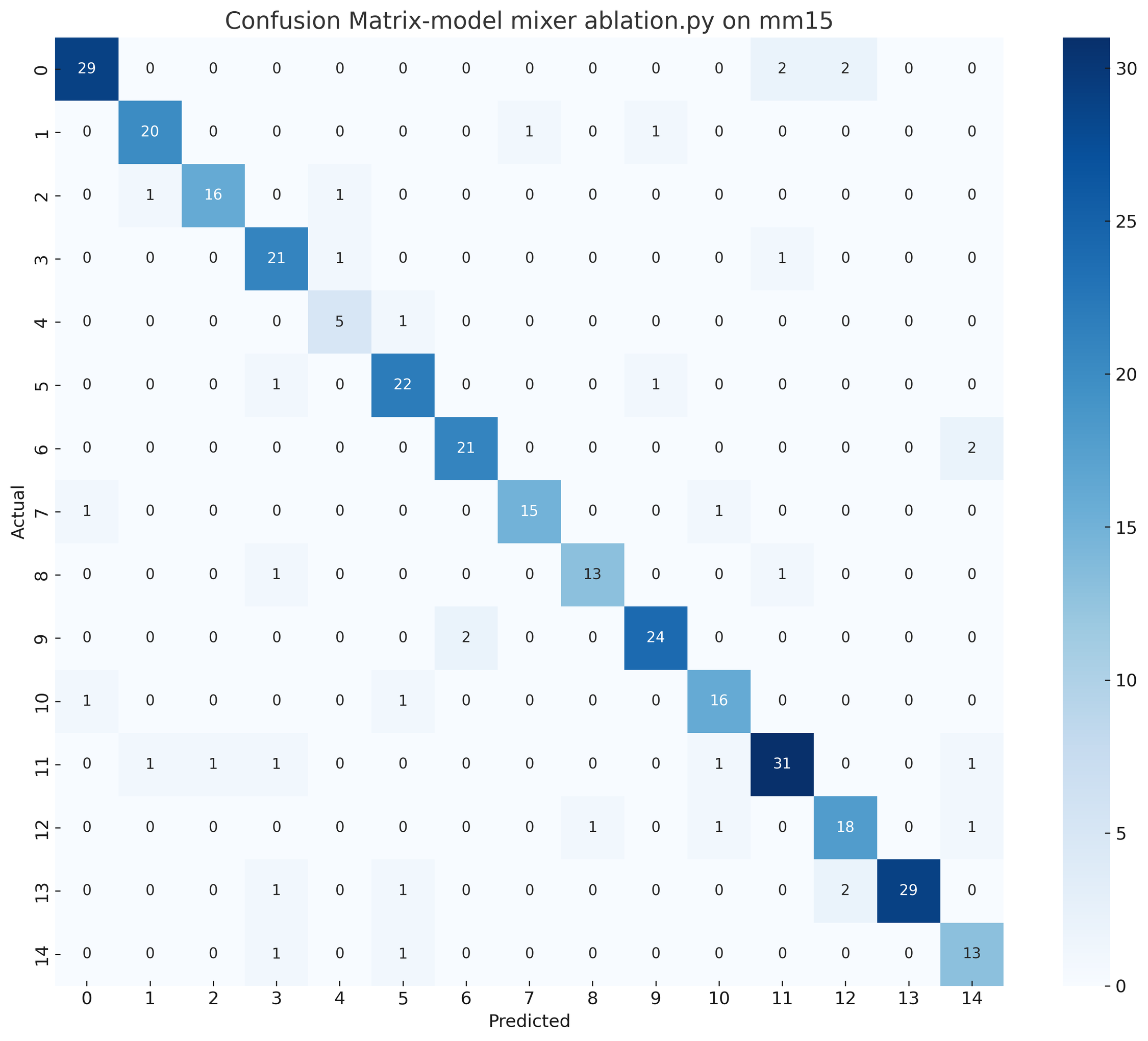

Supplement: S4 Fig — (TIF) [file pone.0330691.s007.tif]

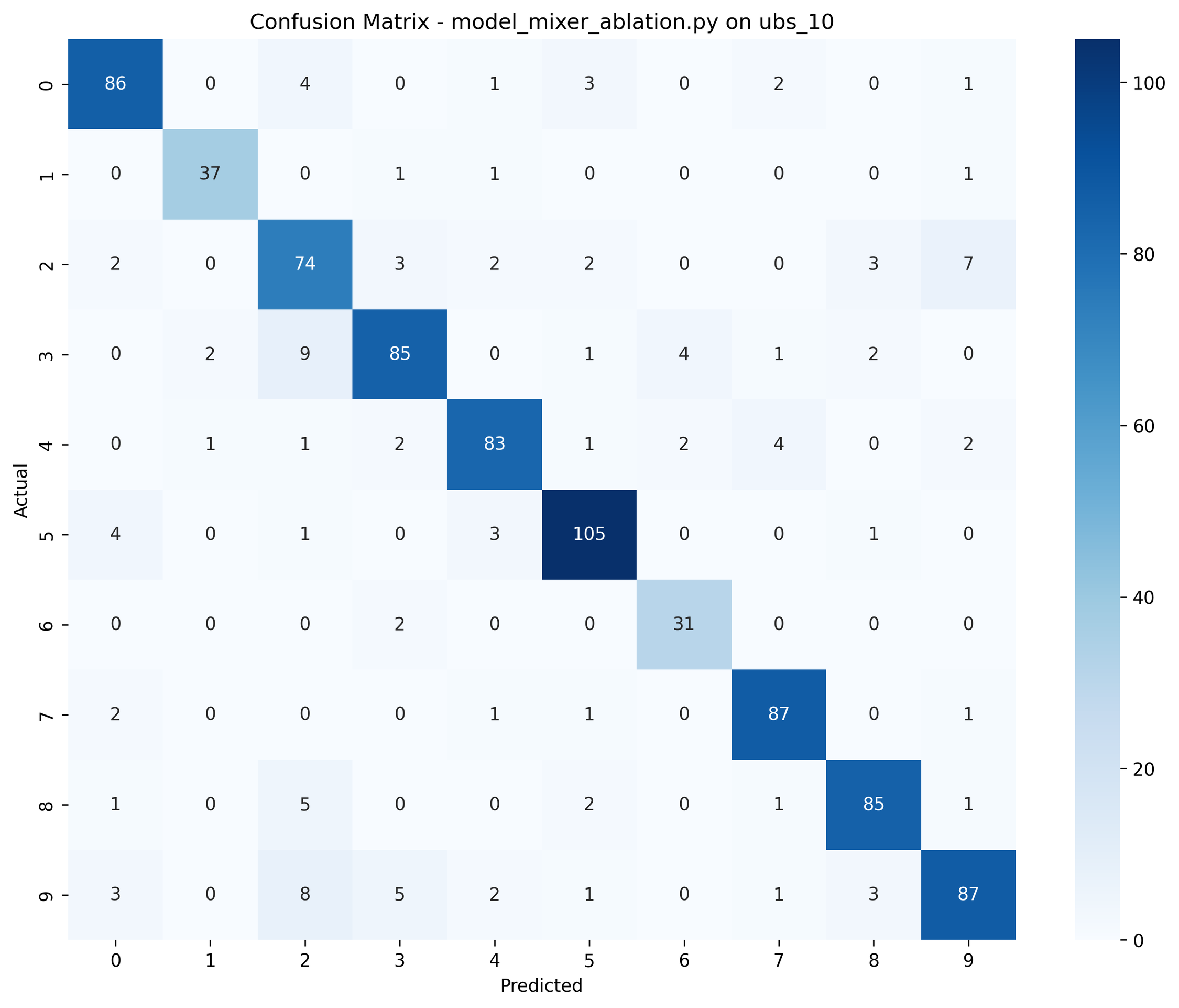

Supplement: S5 Fig — (TIF) [file pone.0330691.s010.tif]

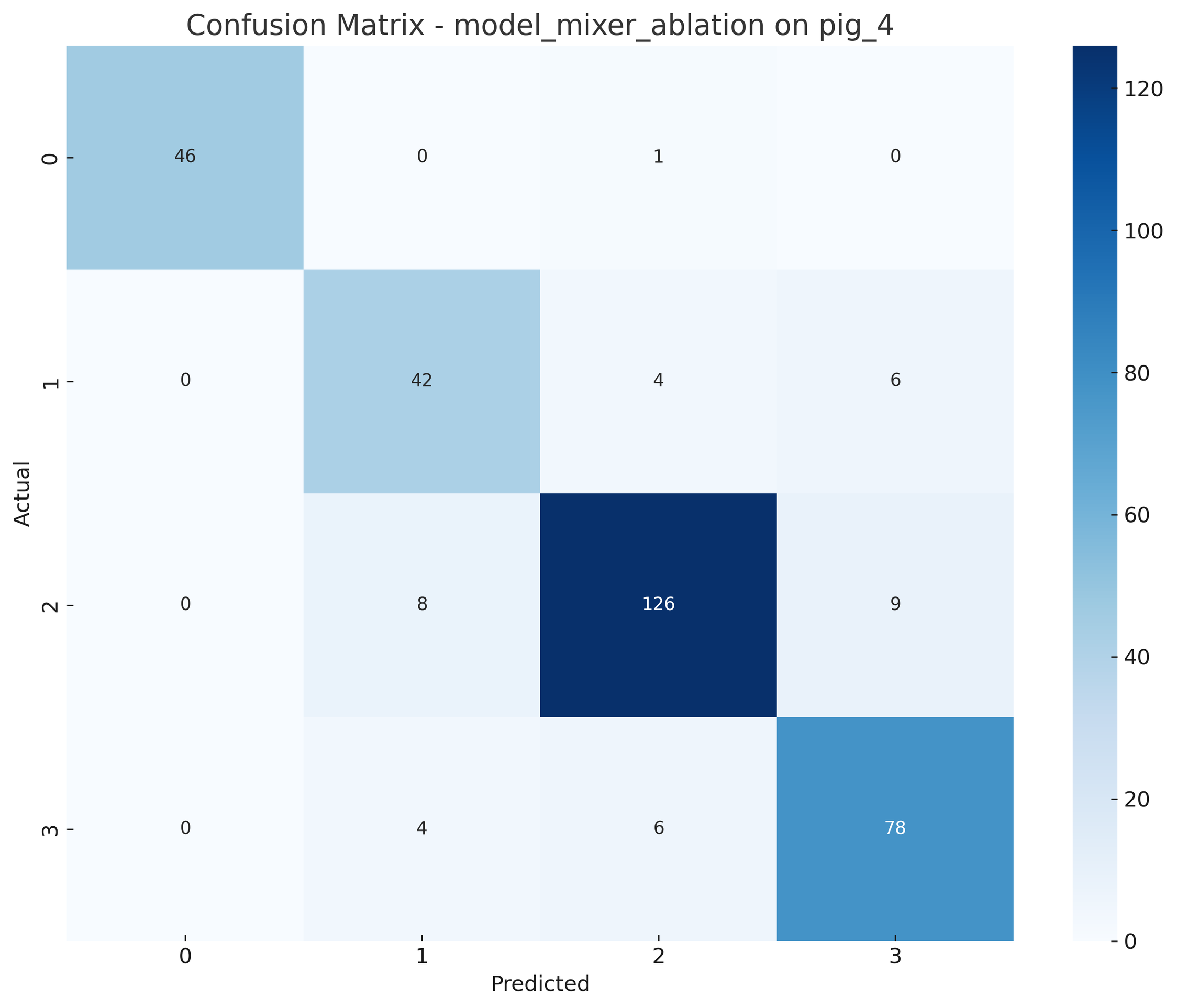

Supplement: S6 Fig — (TIF) [file pone.0330691.s011.tif]
